# Rhabdomyolysis in the Setting of Severe Hyponatremia: A Case Report

**DOI:** 10.7759/cureus.39993

**Published:** 2023-06-05

**Authors:** Diana M Mathew, Akhila Chilakala, Khaled Elfert

**Affiliations:** 1 Internal Medicine, City University of New York (CUNY) School of Medicine, New York City, USA; 2 Internal Medicine, St. Barnabas Hospital Health System, New York City, USA

**Keywords:** psychiatric comorbidities, sodium, electrolyte, rhabdomyolysis, hyponatremia

## Abstract

Electrolyte imbalances are common problems among hospitalized patients, and they can have severe consequences. Although rare, severe hyponatremia or low sodium (Na) levels have been associated with the occurrence of rhabdomyolysis. This is a case of a 45-year-old man who presented with confusion and lethargy and was found to have severe hyponatremia with an elevated creatine phosphokinase (CPK) level of 45,440 IU/L. With the administration of normal saline, the Na levels and CPK improved. He was discharged from the hospital in stable clinical condition. This case demonstrates the need for providers to monitor rhabdomyolysis markers in severe hyponatremia, as there is an observed association between the two and the sequelae can be severe.

## Introduction

Low serum sodium (Na) levels or hyponatremia is a commonly noted electrolyte imbalance and is defined as a serum concentration of less than 135 mEq/L [1]. There are many etiologies of low serum sodium that are further classified by serum osmolality: isotonic, hypotonic, and hypertonic hyponatremia. For isotonic hyponatremia, it is important to rule out hyperproteinuria or hyperlipidemia, as these abnormalities can affect sodium levels. Similarly, hyperglycemia is a common cause of hypertonic hyponatremia. There are many causes of hypotonic hyponatremia: primary polydipsia, syndrome of inappropriate antidiuretic hormone secretion (SIADH), renal failure, adrenal insufficiency, and heart failure. To differentiate between these causes, volume status, urine sodium, and urine osmolality need to be assessed [2,3].

The clinical manifestations of hyponatremia can vary greatly depending on the severity of the deficiency. Some patients are asymptomatic while others can experience seizures, comas, and respiratory arrest. Hyponatremia can less commonly present as rhabdomyolysis, which is characterized by muscle breakdown and the release of intracellular muscle components, such as myoglobin, aldolase, lactate dehydrogenase (LDH), and creatine phosphokinase (CPK) [4-6]. The most common cause of rhabdomyolysis is traumatic injury, but other causes include malignant hyperthermia, drugs, toxins, muscle ischemia, prolonged bed rest, and exertion. Diagnosis is usually made with an elevated CPK level and the condition can also be complicated by renal injury. Though a relatively infrequent finding, associations between rhabdomyolysis and hyponatremia date back to 1979 with a case report of an individual presenting with rhabdomyolysis and myoglobinuria after water intoxication [7].

## Case presentation

A 45-year-old man with schizophrenia, autism spectrum disorder, and generalized anxiety disorder presented to the emergency department with confusion, lethargy, vomiting, and dizziness that began a few hours prior to arrival. He was a disoriented patient and had a difficult time answering most questions, but he verbalized experiencing continuous abdominal pain and headaches. His symptoms had also resulted in a minor fall that caused superficial abrasions to both knees, shoulder, and left forehead. He reported no other significant history. His medical records indicated that his home medications included haloperidol (Haldol®) 10mg QD, hydroxyzine (Atarax®) 25mg QD, clonazepam (Klonopin®) 0.5mg BID, fluoxetine (Prozac®) 10mg QD, pantoprazole (Protonix®) 40mg QD, quetiapine fumarate (Seroquel®) 100mg QD, and benztropine mesylate (Cogentin®) 1mg BID. Medication compliance could not be assessed.

Vital signs on presentation were a blood pressure of 109/59mmHg, heart rate of 81 beats per minute, respiratory rate of 20 breaths per min, temperature was 99.4 degrees Fahrenheit, and oxygen saturation was 98% on room air. Physical exam was unremarkable except for a slight delay in speech with altered mental status and abrasions, as stated above.

His comprehensive metabolic panel and complete blood count on arrival were collected (Tables 1, 2). The complete blood count was measured for 24 hours to trend white blood cell count. The urine toxicology report was positive only for benzodiazepines and no blood alcohol was detected.

**Table 1:** Comprehensive metabolic panel on presentation

	Day 0- 17:33
Sodium (mEq/L)	116
Potassium (mEq/L)	3.3
Chloride (mEq/L)	84
Bicarbonate (mEq/L)	17
Glucose (mg/dL)	115
Blood urea nitrogen (BUN) (mg/dL)	6
Creatinine (mg/dL)	0.8
Calcium (mg/dL)	8.3
Anion gap (mEq/L)	14
Albumin (g/L)	4.2
Total protein (g/dL)	6.3
Alanine transaminase (ALT) (U/L)	41
Aspartate transferase (AST) (U/L)	221
Alkaline phosphatase (ALP) (U/L)	59
Total bilirubin (mg/dL)	2.1

**Table 2:** Complete blood count within first 24 hours MCH - Mean corpuscular hemoglobin, MCHC - mean corpuscular hemoglobin concentration, RDW - red cell distribution width, MPV - mean platelet volume, NRBC - nucleated red blood cell

	Day 0- 17:33	Day 1- 07:45
WBC (10^3/µL)	12.0	5.7
RBC (10^6/µL)	4.11	4.25
Hgb (g/dL)	11.6	12.0
Hct (%)	33.8	36.2
MCV (fL)	82.2	85.2
MCH (pg)	28.2	28.2
MCHC (g/dL)	34.3	33.1
RDW (%)	12.2	13.1
Platelet count	196	171
MPV (fL)	11.7	11.7
Neutrophil (%)	76.9	50.0
Lymphocyte (%)	8.6	28.9
Monocyte (%)	13.3	19.1
Eosinophil (%)	0.2	0.9
Basophil (%)	0.3	0.9
Immature granulocyte (%)	0.7	0.2
NRBC (/100 WB)	0.0	0.0
Neutrophil (10)	9.23	2.86
Lymphocyte (10)	1.03	1.65
Monocyte (10^3)	1.59	1.09
Eosinophil (10)	0.02	0.05
Basophil (10^3)	0.04	0.05
Immature granulocyte (10^3)	0.08	0.01

In the emergency department, the patient was started on 0.9% saline and received 5L over eight hours. While the patient’s symptoms improved, subsequent lab work showed the patient’s sodium levels were overcorrected within 24 hours. The patient’s sodium levels continued to be monitored during his hospital stay (Table 3). His altered mental status improved the morning after arrival; the patient was more compliant, oriented to self, place, and time, and could recall the events leading up to his admission. The patient confirmed that he felt dizzy and fell off the lowest steps of the bus causing the abrasions. He also stated that he had never experienced this before. The patient lives in a supervised group home that administers his medications daily and helps with routine activities.

**Table 3:** Sodium and creatine phosphokinase levels over hospital course CPK- creatine phosphokinase

	Day 0- 17:33	Day 1- 10:29	Day 2- 07:45	Day 3- 04:00	Day 4- 07:00
Na (mEq/L)	116	136	137	137	137
CPK (IU/L)	-	45,440	33,053	-	18,445
Serum Osmolality (mOsm/kg)	-	286	-	-	-
Urine Osmolality (mOsm/kg)	-	124	-	-	-
Urine Sodium (mEq/L)	-	29	-	-	-

To determine the cause of the hyponatremia, the patient’s serum and urine osmolality were measured. CPK levels were checked to determine the extent of trauma. The patient’s CPK peaked within the first 24 hours at 45,440 IU/L. The CPK levels remained elevated but continued to a downtrend until his discharge (Table 3).

## Discussion

Given his psychiatric diagnosis, medication regimen, and the results of the laboratory workup, the hyponatremia in this patient was mostly attributed to primary polydipsia, even though the patient did not provide explicit details about excessive water intake. Other factors could have contributed to the occurrence of hyponatremia in this patient such as medication side effects. Benztropine (an anticholinergic) and hydroxyzine (an antihistaminic with anticholinergic properties) are known to cause dry mouth, potentiating the desire for ingestion of large quantities of water. In addition, neuroleptics and selective serotonin reuptake inhibitors (SSRIs) have also been associated with the development of hyponatremia [8]. SSRIs can cause increased cell permeability through serotonin receptor activation leading to hyponatremia. In the setting of hyponatremia, there is a deficit in the ability to regulate cellular volume and osmolar balance; hence, cell membrane integrity is corrupted which could precipitate cellular injury such as rhabdomyolysis [9-11]. While the exact cause for rhabdomyolysis remains unclear, other changes that affect cellular membrane integrity have been shown to cause increased CPK. For instance, it has been theorized that the sodium-calcium exchange pump in the muscle cells plays an important role. Therefore, in the setting of low serum sodium, the intracellular calcium buildup causes the activation of proteases and lipases, which subsequently cause myocyte lysis [9,10].

Comparably, hyponatremia overcorrection has been known to cause rhabdomyolysis as indicated by elevated CPK and/or renal failure [12]. This degree of elevation can vary generally from the hundreds to the low thousands. This is a less likely cause of the muscle injury in this patient because rhabdomyolysis due to hyponatremia reaches its peak at 48 to 96 hours after while rhabdomyolysis due to hyponatremia overcorrection delays the peak CPK level and is often reached after 96 hours [4]. The peak of the CPK elevation in this patient was on day 1 of admission and 24 hours after the sodium overcorrection so it can be excluded as a cause. Though this could be considered an early peak for hyponatremia-induced rhabdomyolysis, the unclear history makes it difficult to ascertain the correct time interval between the onset of the hyponatremia and the measurement of CPK.

It is important to note that the overcorrection of hyponatremia bears its own risks. Overcorrection is defined as either an increase the sodium levels greater than 8mEq/day in the first 24 hours or greater than 10-12 mEq/L in the first 24 hours and greater than 18 mEq/L in the first 48 hours. Too rapid of an increase in serum Na levels can cause brain dehydration and subsequently lead to osmotic demyelination, which has an incidence as high as 50% in patients with serum Na levels less than or equal to 105 mEq/L [13].

Neuroleptics have also been found to cause a rare adverse drug reaction named neuroleptic malignant syndrome (NMS). It presents with hyperpyrexia (>38°C), muscle rigidity, mental status changes, and autonomic instability such as diaphoresis, tachypnea, tachycardia, urinary incontinence, labile blood pressure, pallor, etc. [14]. This syndrome can also lead to rhabdomyolysis [11]. Though the patient had an altered mental status and had daily haloperidol and fluoxetine use, the patient did not have muscle rigidity, a high fever, or signs of renal damage, which many diagnostic criteria have used as major symptoms for NMS. Due to the varying nature of the criteria, it is important to use NMS as a diagnosis of exclusion [15]. Hyponatremia-induced rhabdomyolysis serves as a better explanation for the patient’s presentation as his symptoms and lab values improved with normal saline and his medications were not discontinued.

Another explanation is that the patient sustained a minor fall that could have contributed to the rise of CPK. Trauma-associated rhabdomyolysis is usually crush injury or motor vehicle accidents which would cause significant muscle injury [5]. The patient’s physical examination did not show extensive injury to cause such high CPK values but it could have contributed slightly. Thus, it is necessary to explore other causes of rhabdomyolysis.

## Conclusions

In summary, physicians should be aware of the rare association between severe hyponatremia and rhabdomyolysis. It is necessary to monitor CPK values because there are many etiologies of rhabdomyolysis especially in patients taking several psychiatric medications and those who have been overcorrected for hyponatremia. Though hyponatremia and the overcorrection of this electrolyte imbalance have their own set of clinical consequences, rhabdomyolysis can have other dangerous effects such as renal injury. Therefore, CPK-level trends should be considered an addition to the guidelines for hyponatremia management. There should be continuing medical education about sodium imbalances and possible manifestations, as well as more research into the pathophysiology behind the association of severe hyponatremia and rhabdomyolysis.
